# The impact of remimazolam sedation during neuraxial anesthesia on perioperative cognitive function in elderly patients: a multicenter randomized controlled study

**DOI:** 10.3389/fphar.2025.1504813

**Published:** 2025-04-28

**Authors:** Dan Qiao, Jia-Min Kang, Rui Zhang, Lin-Yue Zong, Ying Xu, Wei-Wei Zhang, Qi Zhou, Yan Li, Tao Han, Yue-Ming Zhang, Li-Jun Yin, Jin Xu, Shou-Shi Wang, Yuan Yuan, Qing Li, Kai-Jun Niu, Yu-Xin Zheng, Lin-Lin Zhang, Yi-Ze Li, Yong-Hao Yu

**Affiliations:** ^1^ Department of Anesthesiology, Tianjin Medical University General Hospital, Tianjin Research Institute of Anesthesiology, Tianjin, China; ^2^ Department of Anesthesiology, Shanxi Provincial People’s Hospital, Taiyuan, Shanxi, China; ^3^ Department of Anesthesiology, Chifeng Municipal Hospital, Chifeng, China; ^4^ Department of Anesthesiology, Tianjin Jizhou People’s Hospital, Tianjin, China; ^5^ Department of Anesthesiology, Weifang People’s Hospital, Weifang, Shandong, China; ^6^ Department of Anesthesiology, Tianjin Baodi Hospital, Tianjin, China; ^7^ The Second Department of Anesthesiology, Tianjin Hospital, Tianjin, China; ^8^ Department of Anesthesiology, Central Hospital Affiliated to Qingdao University, Qingdao, China; ^9^ Nutritional Epidemiology Institute and School of Public Health, Tianjin Medical University, Tianjin, China; ^10^ Yantai Affiliated Hospital of Binzhou Medical University, Yantai, China

**Keywords:** remimazolam, sedation, neuraxial anesthesia, postoperative cognitive complications, cognitive function, elderly

## Abstract

**Background:**

Remimazolam, a novel ultra-short-acting benzodiazepine, is a potential sedative for non-general anesthesia surgery in the elderly. This study aimed to investigate the appropriate sedative dosage of remimazolam and its effects on perioperative cognitive function in elderly patients undergoing non-general anesthesia surgery.

**Methods:**

This multicenter, placebo-controlled trial enrolled 330 elderly patients undergoing non-general anesthesia procedures at eight centers in China from July 2021 to February 2022, with 238 ultimately completing the study. The primary endpoints were the dose of successful sedation with remimazolam and the changes in perioperative cognitive function. Adverse events were recorded to assess drug safety.

**Results:**

The induction dose of remimazolam for sedation in spinal anesthesia in elderly patients was 5.38 mg (95% confidence interval [CI], 5.20–5.56), maintained at a rate of 0.223 mg·kg^−1^·h^−1^ (95% CI, 0.201–0.237) with no serious adverse effects. Compared with the standard saline group, there was no statistical difference in the MMSE scores on Day 2 morning (P = 0.886), Day 2 afternoon (P = 0.864), and Day 7 (P = 0.613), and no statistical difference in the MoCA scores on Day 2 morning (P = 0.687), Day 2 afternoon (P = 0.827), and Day 7 (P = 0.483) in remimazolam group.

**Conclusion:**

Remimazolam besylate is an effective sedative for elderly patients undergoing neuraxial anesthesia. It was successfully induced at a dose of 5.38 mg and maintained at 0.223 mg·kg^−1^·h^−1^, demonstrating a good safety profile without affecting short-term postoperative cognitive function.

**Clinical Trial Registration:**

http://www.chictr.org.cn (ChiCTR2100048744).

## 1 Introduction

The aging of the population presents significant challenges to public health systems (Jensen et al., 2020). Elderly patients face an elevated risk of perioperative organ damage due to progressive decline in organ function (Olotu, 2021; Lim and Lee, 2020; Lee et al., 2021). In comparison to general anesthesia, spinal and local anesthesia require fewer drugs, reducing the accumulation of anesthetics and minimizing their impact on cardiac and pulmonary perfusion (Desai et al., 2018; Liu et al., 2014; Mounet et al., 2021). However, patients who remain awake during surgery are more likely to experience tension, anxiety, and fear, which may result in stress and elevate the risk of cardiovascular and cerebrovascular complications, as well as postoperative cognitive dysfunction (Urvoy et al., 2021). Postoperative delirium (POD) is a common and serious complication in the elderly following surgery. It involves a decline in neurocognitive function after anesthesia and surgery, representing a central nervous system complication (Rundshagen, 2014). POD is characterized by a marked reduction in cognitive abilities, such as memory, attention, coordination, orientation, verbal fluency, and executive function (Needham et al., 2017). POD is generally believed to result from a combination of factors, including patient age, type of surgery and anesthesia, pain severity, and educational background (Zhao et al., 2024). POD may persist for weeks to years, negatively adversely affecting recovery, prolonging hospital stays, and contributing to additional physical and mental health complications. It also elevates mortality rates and places a substantial burden on both patients and their families (Gu et al., 2019; Mason et al., 2010; Miller et al., 2018; Skvarc et al., 2018; Xiao et al., 2020). Recent studies have highlighted the important role of exosomes in regulating neuroinflammation and neuroprotection. Exosomes are nano-sized extracellular vesicles that carry bioactive molecules such as proteins, mRNAs, and miRNAs, and have been shown to modulate β-amyloid (Aβ) metabolism and reduce neuroinflammatory responses (Hosseini and Abolhasanpour, 2001). The etiology of POD is complex, and its exact mechanism remains unclear. However, the neuroinflammatory response triggered by surgery is widely regarded as the primary contributing factor and initial trigger in its development (Skvarc et al., 2018).

Commonly used sedatives include midazolam, propofol, and dexmedetomidine, each associated with specific safety concerns (Tran et al., 2019). Midazolam, for instance, is linked to an increased the risk of postoperative delirium (Aldecoa et al., 2017; Fick et al., 2019), while propofol may significantly affect hemodynamics (Fechner et al., 2024), and dexmedetomidine may cause transient bradycardia and hypertension (Coursin et al., 2001). Remimazolam, a novel ultra-short-acting benzodiazepine, undergoes rapid metabolized by nonspecific esterase into the inactive metabolite CNS7054. It offers rapid onset of action, a shorter half-life than midazolam, favorable circulatory and respiratory stability, and can be immediately antagonized by flumazenil (Sheng et al., 2020; Sneyd and Rigby-Jones, 2020). Clinical trials have shown that remimazolam provides sedation efficacy comparable to propofol for procedures like gastroscopy and hysteroscopy, with the added benefits of painless injection, minimal respiratory depression, and no adverse effects on short-term postoperative cognitive function (Doi et al., 2020; Guan et al., 2021; Tan et al., 2022; Zhang et al., 2022; Zhang et al., 2021). Moreover, remimazolam has been demonstrated to alleviate neuropathic pain, reduce the production of pro-inflammatory factor production such as TNF-α, IFN-γ, IL-6, IL-1β (Xie et al., 2021), and mitigate cerebral ischemia/reperfusion (I/R) injury (Shi et al., 2022). For elderly patients undergoing surgery with neuraxial anesthesia, the requirements for sedatives are relatively high. Additionally, since patients do not completely lose consciousness as they would under general anesthesia, they often experience heightened anxiety during the procedure. An ideal sedative should not only provide a comfortable diagnostic and therapeutic experience but also exhibit rapid onset, quick recovery after discontinuation, predictable dose-response relationships, no accumulation, and minimal adverse effects. In summary, we believe that remimazolam holds significant promise for use in elderly patients receiving neuraxial anesthesia. Despite its ability to induce and maintain general anesthesia, there are currently no reports sedation regimens using remimazolam during neuraxial anesthesia in elderly patients.

This study, therefore, aims to identify the optimal sedation dose of remimazolam for neuraxial anesthesia and to evaluate its potential impact on perioperative cognitive function.

## 2 Materials and methods

### 2.1 Ethical approval and study population

This study was a prospective, multicenter, randomized, single-blind, placebo-controlled clinical trial approved by the Medical Ethics Committee of Tianjin Medical University General Hospital (Approval number: IRB2021-YX-067–01). Registration was completed via the Chictr.org.cn registration system (Registration number: ChiCTR2100048744). Written informed consent was obtained from all participants or their families in accordance with CONSORT guidelines. A total of 330 older patients (American Society of Anesthesiologists physical status I–III,age ≥65 years) were recruited from eight centers in China for neuraxial anesthesia procedures between July 2021 and February 2022. Surgical procedures included cystoscopy, transurethral resection of the prostate (TURP), hemorrhoidectomy, surgical amputation, total knee arthroplasty (TKA), and partial knee replacement (PKR). The eight participating centers included Tianjin Medical University General Hospital, Shanxi Provincial People’s Hospital, Chifeng Municipal Hospital, Tianjin Jizhou People’s Hospital, Weifang People’s Hospital, Tianjin Baodi Hospital, Tianjin Hospital, and Central Hospital Affiliated with Qingdao University. Exclusion criteria included allergies to any anesthetic drug, contraindications to neuraxial anesthesia, severe respiratory pathology, psychiatric disorders, cognitive dysfunction (MoCA score <26 points), pregnancy, or breastfeeding.

### 2.2 Randomization and masking

All eligible patients were randomized through a grouping system managed by the School of Public Health, Tianjin Medical University. Patients were divided into a remimazolam group (R group) and a 0.9% sodium chloride injection group (saline group) in a 2:1 ratio. Given the significant differences in sedation efficacy and appearance between the two groups, a single-blind design was utilized. As a result, the attending anesthesiologist could not be blinded. However, study participants, post-anesthesia care unit nurses, and statisticians remained blinded to the trial subgroups.

### 2.3 Procedures

A professionally trained fellow conducted preoperative assessments of potential enrollees within 7 days before surgery (days 7–1), evaluating general condition, sleep rhythm (Pittsburgh Sleep Quality Index, PSQI), anxiety level (Hamilton Anxiety Scale, HAMA), delirium condition (Nursing Delirium Screening Scale, Nu-DESC), cognitive function (Mini-Mental State Examination [MMSE] and Montreal Cognitive Assessment [MoCA]).

Upon admission to the operating room (Day 1), patients were routinely monitored for electrocardiogram, blood pressure, oxygen saturation, temperature, and bispectral index (BIS). Based on previous studies evaluating sedative drugs with BIS monitoring (Bae et al., 2024; Zhao et al., 2023), sedation levels were assessed using both the Modified Observer Alertness/Sedation Assessment (MOAA/S) scale and the BIS index.

Sedation was initiated after the completion neuraxial anesthesia (epidural, lumbar, and combined lumbar and epidural anesthesia) to ensure sufficient anesthesia for surgery. Patients in the remimazolam group received an initial dose of remimazolam besylate (5.0 mg), while the saline group received 5 mL of 0.9% saline as a placebo. If patients in the remimazolam group had a MOAA/S score ≥3 at 1∼3 min after the initial dose, additional intravenous doses (2.5 mg per dose at 2-min intervals) were administered until the MOAA/S score dropped to ≤2. Sedation was considered unsuccessful if more than five additional doses (beyond the initial dose) were required during induction and the MOAA/S score remained >4.

Maintenance dosing was initiated once sedation was considered sufficient (MOAA/S score ≤2). In the remimazolam group, remimazolam besylate was continuously administered at 0.2 mg·kg^−1^·h^−1^, while the saline group received 0.9% saline at 0.2 mL·kg^−1^·h^−1^. The maintenance dosing rate was adjusted according to the patient’s MOAA/S score, with increments or decrements of 0.1 mg·kg^−1^·h^−1^ as necessary. Additional doses of 2.5 mg of remimazolam besylate were administered as needed.

If the patient’s mean arterial pressure (MAP) and heart rate (HR) decrease by more than 20% from the preoperative baseline or systolic blood pressure fell to ≤80 mmHg, fluid therapy or vasoactive drugs were administered immediately. If the patient’s oxygen saturation dropped below 90%, mask-assisted ventilation was immediately initiated, with laryngeal mask or endotracheal intubation performed if necessary.

**FIGURE 1:**
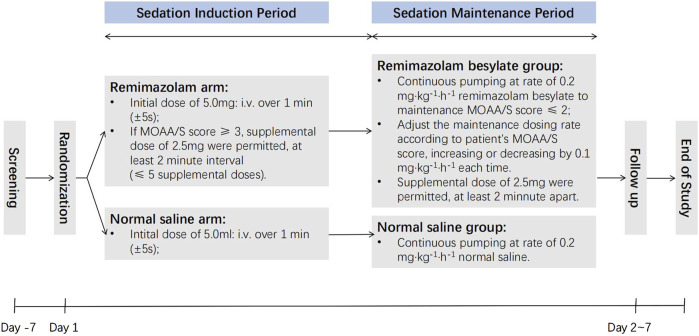
The flowchart of study.

Investigators assessed patients’ cognitive function, sleep rhythm, anxiety, delirium, and fragility on both the morning and afternoon of Day 2 and on Day 7. Patient and investigator satisfaction levels were evaluated as well. Satisfaction was rated on a scale from 0 to 10, with 0∼3 categorized as poor, 4∼7 as fair, and 8∼10 as excellent. A flowchart illustrating the study design is provided in Figure 1.

### 2.4 Statistical analysis

This trial employed a blank placebo as a control, with sample size calculation was based on relevant literatures (Zhang et al., 2021; Kornstein et al., 2014; Rex et al., 2018; Rex et al., 2021; Pambianco et al., 2016). A sedation success rate of 93% was assumed for both the propofol and remimazolam besylate groups with a 2:1 ratio between the trial and saline groups. Therefore, 176 participants were required for the remimazolam group and 88 in the saline group (α = 0.025, one-sided, and power 80%). Considering a 20% loss to follow-up, the target enrollment was set at 220 patients in the remimazolam group and 110 in the saline group, for a total of 330 patients.

Statistical analyses were conducted using GraphPad Prism 9.5. Statistical significance was defined as P < 0.05, with a 95% confidence interval (CI). Continuous data were presented as mean ± standard deviation, median, minimum, and maximum values. Within-group changes from baseline were analyzed using a paired t-test, while differences between groups before and after treatment were analyzed using Analysis of Variance (ANOVA) or non-parametric tests. Categorical data were summarized using frequency and percentage, and differences before and after treatment were evaluated using the χ^2^ test, Fisher’s exact method, or non-parametric tests.

## 3 Results

**FIGURE 2:**
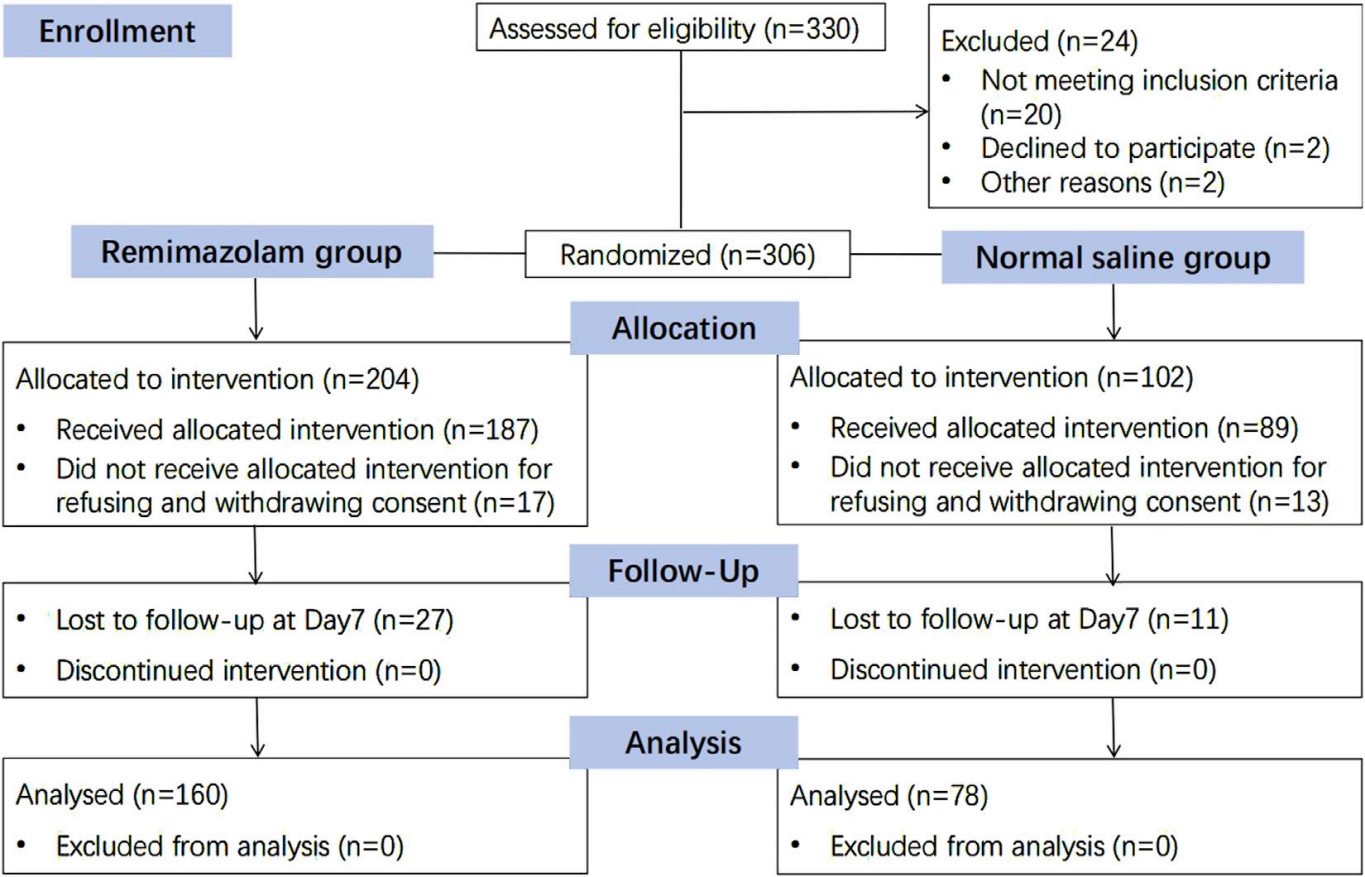
Consolidated standards of reporting trials (CONSORT) diagram.

A total of 330 patients were recruited between July 2021 and February 2022. Eligible patients were randomized to receive either remimazolam or placebo sedation in a 2:1 ratio. Of the recruited participants, 21 refused to participate, 9 withdrew consent on the day of surgery, and 38 declined postoperative follow-up, leaving 238 patients for analysis (Figure 2). The two groups were comparable in terms of demographic and baseline characteristics (Table 1).

**TABLE 1:** Patient and anesthetic characteristics.

Characteristics	Remimazolam (n = 160)	Saline (n = 78)	P
Sex			
Male	73 (46%)	40 (51%)	0.195
Female	87 (54%)	38 (49%)	0.359
Age (yr)	69.9 ± 4.7	69.9 ± 4.5	0.463
Body weight (kg)	66.7 ± 1 0.9	67.5 ± 9.9	0.413
BMI (kg·m^2^)	24.3 ± 2.26	24.2 ± 2.31	0.860
ASA physical status			0.623
2	142 (89%)	74 (95%)	
3	18 (11%)	4 (5%)	
Preoperative comorbidities			
Stroke	1	0	0.687
Hypertension	67	24	0.175
Coronary heart disease	38	19	0.471
Arrhythmia	0	0	—
COPD	7	1	0.668
Chronic bronchitis	0	0	—
Asthma	0	0	—
Diabetes	13	5	0.640
Liver dysfunction	11	4	0.645
Renal dysfunction	2	0	0.685
MAP (mmHg)	95.4 ± 7.2	97.2 ± 8.0	0.177
HR (beats·min^−1^)	76.1 ± 7.5	76.4 ± 7.6	0.725
Surgery history, n	44	35	
Duration of Sedation (min)	74.0 ± 22.0	69.5 ± 17.4	0.068
Duration of Surgery (min)	77.5 ± 21.0	74.1 ± 18.0	0.084

Data are mean ± SD, or n (%).

ASA, american society of anesthesiologists; BMI, body mass index; COPD, chronic obstructive pulmonary disease; Liver dysfunction is defined as serum alanine and/or aspartate transaminase higher than five times the upper normal limit; Renal dysfunction is defined as Creatinine concentration higher than 177 μmol/L.

### 3.1 The primary outcome: dose of effective sedation

The sedation effects in both groups are presented in Table 2. Sedation failed in 2 of the 187 patients who received remimazolam, yielding a sedation success rate of 98.93%; however, both patients declined postoperative follow-up. In the remimazolam group, the average initial dose was 5.38 mg (95% CI, 5.20–5.56), and the average time to achieve successful sedation (MOAA/S ≤ 2) was 1.61 min (95% CI, 1.40–1.81). The mean maintenance dose was 0.223 mg·kg^-1^·h^-1^ (95% CI, 0.201–0.237), and the mean time to reach a MOAA/S score of 5 during recovery was 8.03 min (95% CI, 7.68–8.37) after drug discontinuation.

**TABLE 2:** Dose of successful sedation.

Outcome	Remimazolam (n = 160)	Saline (n = 78)
Sedation success time (min)	1.61 (1.40–1.81)	—
Remimazolam induction dose (mg)	5.38 (5.20–5.56)	—
Remimazolam maintenance dose (mg·kg^−1^·h^−1^)	0.22 (0.20–0.24)	—
Total dose of remimazolam per patient (mg)	23.50 (21.94–25.06)	—
Wake-up time (min)	8.03 (7.68–8.37)	—

Unless specified, all values are expressed as mean (95%).

### 3.2 The secondary outcomes: changes in cognitive function

Perioperative cognitive function changes in both groups are shown in Table 3. No significant differences were found between the two groups in MMSE and MoCA scores at any time point (p > 0.05). Specifically, the preoperative baseline MMSE (P = 0.848) and MoCA scores (P = 0.956) were comparable between the groups. Compared with the saline group, the MMSE scores did not significantly differ in the remimazolam group on Day 2 morning (mean ± SD, 24.49 ± 0.22 vs. 24.54 ± 0.24; 95% CI, −0.75 to 0.65; P = 0.886), Day 2 afternoon (mean ± SD, 24.55 ± 0.22 vs. 24.49 ± 0.26; 95% CI, −0.66 to 0.78; P = 0.864), or on Day 7 (mean ± SD, 24.61 ± 0.22 vs. 24.42 ± 0.26; 95% CI, −0.53 to 0.90; P = 0.613), similarly, MoCA scores showed no significant differences on the morning (mean ± SD, 21.03 ± 0.29 vs. 21.05 ± 0.34; 95% CI, −0.97 to 0.92; P = 0.956) or afternoon (mean ± SD, 21.23 ± 0.29 vs. 21.04 ± 0.34; 95% CI, −0.75 to 1.13; P = 0.687) of Day 2. T-MoCA scores were also similar between the two groups during the telephone follow-up on postoperative Day 6 (Day 7, mean ± SD, 14.41 ± 0.34 vs. 14.05 ± 0.31; 95% CI, −0.64 to 1.35; P = 0.483).

**TABLE 3:** Changes in cognitive function.

Outcome	Remimazolam (n = 160)	Saline (n = 78)	P
MMSE score (Preoperative)	24.59 ± 0.22	24.53 ± 0.25	0.848
MMSE score (Day 2 morning)	24.49 ± 0.22	24.54 ± 0.24	0.886
Change from Preoperative score	−0.11 ± 0.05	0.01 ± 0.06	0.136
Score <26	93 (58.1%)	49 (62.8%)	0.079
MMSE score (Day 2 afternoon)	24.55 ± 0.22	24.49 ± 0.26	0.864
Change from Preoperative score	−0.4 ± 0.05	0.04 ± 0.06	0.948
Score <26	92 (57.5%)	51 (65.4%)	0.105
MMSE score (Day 7)	24.61 ± 0.22	24.42 ± 0.26	0.613
Change from Preoperative score	0.01 ± 0.04	−0.01 ± 0.06	0.092
Score <26	89 (55.6%)	51 (65.4%)	0.107
MoCA score (Preoperative)	21.03 ± 0.29	21.05 ± 0.34	0.956
MoCA score (Day 2 morning)	21.23 ± 0.29	21.04 ± 0.34	0.687
Change from Preoperative score	0.27 ± 0.06	0.22 ± 0.10	0.654
MoCA score (Day 2 afternoon)	21.36 ± 0.29	21.26 ± 0.35	0.827
Change from Preoperative score	0.34 ± 0.07	0.21 ± 0.09	0.261
T-MoCA score (Day 7)	14.41 ± 0.34	14.05 ± 0.31	0.483

Data are n (%) or mean ± SD. CI, confidence interval; MMSE, Mini-Mental State Examination; MoCA, Montreal Cognitive Assessment. T-MoCA, Telephone-MoCA.

Therefore, it was concluded that remimazolam benzodiazepine use in elderly patients undergoing neuraxial anesthesia does not increase the incidence of postoperative cognitive dysfunction.

### 3.3 Change in delirium, sleep and anxiety

Perioperative delirium, anxiety, and sleep for both groups are shown in Table 4. No statistically significant differences were observed between the two groups in preoperative baseline Nu-DESC, HAMA, and PSQI scores. Compared to baseline values, the delirium scores (Nu-DESC) in both groups showed no significant changes on the day of surgery (Day 1) (P = 0.153), Day 2 morning (P = 0.817), Day 2 afternoon (P = 0.486), or on Day 7 (P = 0.787). No significant differences in delirium scores (Nu-DESC) were observed between the two groups on Day 1 (P = 0.697), the morning (P = 0.850) and afternoon (P = 0.549) of Day 2, or on Day 7 (P = 0.604).

**TABLE 4:** Change in delirium, sleep and anxiety.

Outcome	Remimazolam (n = 160)	Saline (n = 78)	P-value
Nu-DESC score (Preoperative)	0.03 ± 0.02	0.03 ± 0.03	0.982
Nu-DESC score (Day 1)	0.03 ± 0.02	0.04 ± 0.04	0.697
Change from Preoperative score	0 ± 0	0.01 ± 0.01	0.153
Nu-DESC score (Day 2 morning)	0.03 ± 0.02	0.03 ± 0.03	0.850
Change from Preoperative score	0.01 ± 0.02	0 ± 0	0.817
Nu-DESC score (Day 2 afternoon)	0.01 ± 0.01	0.03 ± 0.03	0.549
Change from Preoperative score	−0.01 ± 0.01	0 ± 0	0.486
Nu-DESC score (Day 7)	0.01 ± 0.01	0.01 ± 0.01	0.604
Change from Preoperative score	−0.02 ± 0.01	−0.01 ± 0.01	0.787
HAMA score (Preoperative)	5.33 ± 0.31	5.28 ± 0.38	0.934
Score ≥7	70 (43.8%)	32 (41.0%)	0.240
HAMA score (Day 2 morning)	1.82 ± 0.20	4.93 ± 0.35	**< 0.001**
Change from Preoperative score	−3.51 ± 0.19	−0.35 ± 0.13	**< 0.001**
Score ≥7	18 (11.3%)	32 (41.0%)	0.101
HAMA score (Day 2 afternoon)	1.18 ± 0.16	4.33 ± 0.35	**< 0.001**
Change from Preoperative score	−4.15 ± 0.22	−0.95 ± 0.19	**< 0.001**
Score ≥7	11 (6.9%)	32 (41.0%)	0.182
HAMA score (Day 7)	0.69 ± 0.12	3.51 ± 0.36	**< 0.001**
Change from Preoperative score	−4.63 ± 0.25	−1.77 ± 0.25	**< 0.001**
Score ≥7	3 (1.88%)	26 (33.3%)	0.235
PSQI score (Preoperative)	5.06 ± 0.16	5.10 ± 0.18	0.862
Score ≥8	13 (8.13%)	9 (11.54%)	0.078
PSQI score (Day 2 morning)	4.74 ± 0.17	5.01 ± 0.16	0.301
Change from Preoperative score	−0.32 ± 0.09	−0.09 ± 0.11	0.135
Score ≥8	11 (6.88%)	6 (7.69%)	0.255
PSQI score (Day 7)	4.32 ± 0.18	5.35 ± 0.14	**0.002**
Change from Preoperative score	−0.72 ± 0.12	0.24 ± 0.13	**<0.001**
Score ≥8	9 (8.49%)	5 (6.41%)	0.427

Data are n (%) or mean ± SD. CI, confidence interval; Nu-DESC, nursing screening scale; HAMA, hamilton anxiety scale; PSQI, pittsburgh sleep quality index. The bolded P value indicates significant statistical significance.

Compared to preoperative values, the HAMA scores decreased significantly on Day 2 morning (P < 0.001), Day 2 afternoon (P < 0.001), as well as on Day 7 (P < 0.001) in both groups. Significant differences in HAMA scores between the two groups were also observed on the morning (P < 0.001) and afternoon (P < 0.001) of Day 2 and on Day 7 (P < 0.001). However, anxiety was diagnosed only when HAMA scores were ≥7 (Pastis et al., 2022), and there were no significant differences in the degree of perioperative anxiety between the two groups.

In terms of sleep quality (Pittsburgh Sleep Quality Index [PSQI]), no statistically significant differences were observed between the two groups for all patients (P = 0.862) or for those with sleep disorders (PSQI ≥8) (P = 0.078). On morning of day 2, the difference in PSQI scores between the groups (P = 0.301) and the change from preoperative baseline scores (P = 0.135) was not statistically significant. However, significant differences in PSQI scores and changes from baseline (P < 0.001) were observed between the groups on postoperative Day 7.

We conclude that an appropriate dose of remimazolam benzodiazepine during neuraxial anesthesia is unlikely to cause postoperative adverse effects such as delirium, sleep disorders or anxiety.

### 3.4 Postoperative satisfaction

The patient and researcher satisfaction scores for the two groups are shown in Supplementary Table S1. On both the morning and afternoon of Day 2, as well as Day 7, patient and researcher satisfaction in the remimazolam group was significantly higher than in the saline group. The differences were statistically significant (All P < 0.001).

### 3.5 The correlation between BIS and MOAA/S in the remimazolam group

**FIGURE 3:**
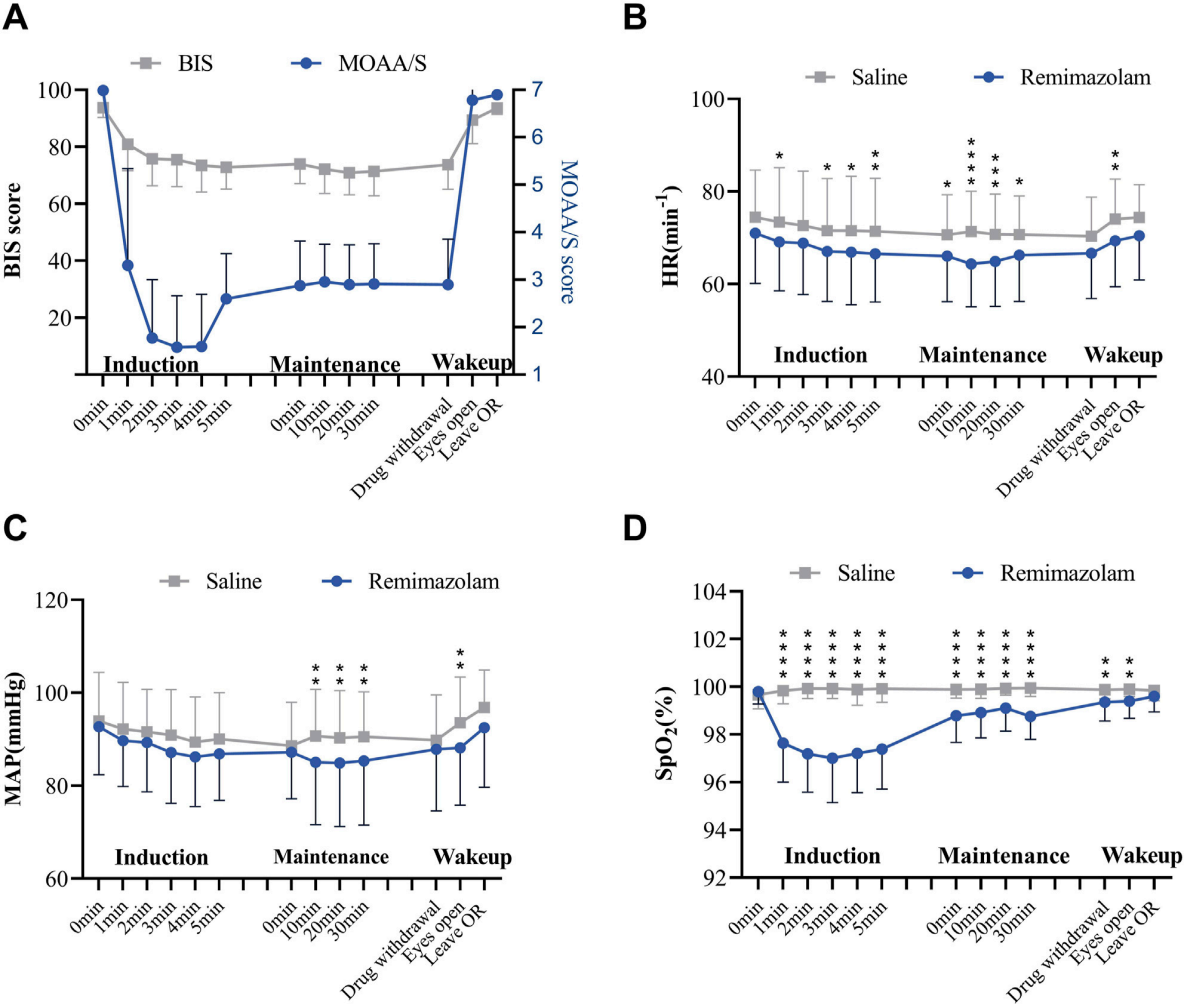
The effects of remimazolam sedation on patients’ vital signs. **(A)** Mean arterial pressure, **(B)** heart rate, **(C)** SpO_2_ and **(D)** BIS index and MOAA/S scale change during remimazolam sedation. Mean ± SD; *p < 0.05, **p < 0.01, ***p < 0.001, ****p < 0.0001; n = 78 in saline group, n = 160 in remimazolam group; 2-way ANOVA followed by post-hoc analysis with Bonferroni corrections.

We monitored the BIS index and MOAA/S scale simultaneously during procedures performed on the patients in the remimazolam group (Figure 3A). During the sedation maintenance period, BIS values consistently exceeded 70 (mean ± SD:71.00 ± 7.78 to 75.79 ± 9.47), which was markedly different from the actual sedation state. However, the MOAA/S score decreased rapidly to <2 approximately 2 minutes after the start of induction, which was generally consistent with the actual sedation level of the patients. The results indicated that BIS values did not correlate with MOAA/S scores at corresponding time points, whereas the MOAA/S score strongly correlated with sedation depth in the remimazolam group.

### 3.6 Safety analysis

The MAP and HR of patients in the remimazolam group decreased within the first 5 minutes after the sedation induction period. Both MAP and HR values were lower than those in the saline group; however, all heart rate values remained within the safe range, and this effect has a cardioprotective impact on patients (Figures 3B,C). Patients experienced a mild decrease in SpO_2_ after induction of remimazolam sedation, but SpO_2_ levels recovered gradually and remained above 95% (Figure 3D). Therefore, the sedation with remimazolam in neuraxial anesthesia surgery had no significant impact on vital signs but reduced heart rate and mean arterial pressure, suggesting decreased perioperative oxygen consumption in patients.

### 3.7 Incidence of adverse reactions to anesthesia

The use of remimazolam did not increase the number of doses of phenylephrine (P = 0.236), atropine (P = 0.619), dopamine (P = 0.291), metaraminol bitrate (P = 0.971), methoxamine (P = 0.564), or ephedrine (P = 0.0.291) compared with that in the saline group. In terms of adverse effects, the incidences rates of nausea (P = 0.983), vomiting (P = 0.602), drowsiness (P = 0.321), headache (P = 0.484), dizziness (P = 0.321), and gloss coma (P = 0.321) were not significantly higher in the remimazolam group. However, the incidence of chills was significantly reduced (P < 0.001) in this group. (Supplementary Table S2). This result suggests that remimazolam has no obvious side effects and has an important preventive effect in reducing the increase in oxygen consumption and patient discomfort caused by shivering.

## 4 Discussion

The main results of this multicenter trial are as follows: (i) the effective sedation dose of remimazolam besylate in elderly patients under neuraxial anesthesia was determined to be 5.38 mg. The appropriate induction dosing regimen consisted of an initial dose of 5 mg, a supplemental dose of 2.5 mg, and a maintenance infusion rate of 0.223 mg·kg^−1^·h^−1^; (ii) remimazolam besylate did not impair short-term postoperative cognitive function in elderly patients.

Unlike midazolam, remimazolam besylate has distinct pharmacodynamic and pharmacokinetic properties, making it suitable for maintaining sedation during short procedures, such as gastrointestinal endoscopy, hysteroscopy, and fiberoptic bronchoscopy (Zhang et al., 2022; Zhang et al., 2021; Kornstein et al., 2014; Wesolowski et al., 2016). Remimazolam exerts minimal effects on the circulatory and respiratory systems, can be rapidly antagonized by flumazenil (Nakayama et al., 2021), and is particularly suitable for elderly patients. Notably, the 0.1 mg·kg^−1^ remimazolam dose in elderly patients undergoing upper gastrointestinal endoscopy did not affect short-term postoperative cognitive function (Tan et al., 2022). Remimazolam is relatively safe for both induction (1 mg·kg^−1^·h^−1^) and maintenance (0.1 mg·kg^−1^·h^−1^) in elderly patients undergoing general anesthesia (Chen et al., 2021; Nakanishi et al., 2021). However, specific guideline dosing regimens for remimazolam besylate for sedation during neuraxial anesthesia in elderly patients have not yet been established.

Based on prior studies (Doi et al., 2020; Rex et al., 2018) and the manufacturer’s recommendations, we selected a titrated dosing regimen for induction and an individualized dosing regimen for maintenance. In our trial, two cases of sedation failure led to a sedation efficiency of 98.93%, consistent with the results of a Phase III clinical trial in China (Zhao et al., 2022). Another study reported effective sedation in elderly male patients with a single dose of remimazolam besylate, achieving a 50% effective dose (ED_50_) of 0.063 mg·kg^−1^ and a 95% effective dose (ED_95_) of 0.079 mg·kg^−1^ (Ichijima et al., 2022). In some cases, the effective dose required for successful sedation may be lower than 5.38 mg with this regimen. This discrepancy may stem from our definition of successful sedation as MOAA/S ≤ 2, in contrast to MOAA/S ≤ 3 in the other study. Our trial’s median age was 69.9 years, with a median Body Mass Index (BMI) of 24.0 kg·m^−2^ and a balanced gender distribution. In contrast, the other study only included male patients with a mean BMI of 21.87 kg·m^−2^ and a mean age of 74.8 years.

Another finding of our trial was that, unlike midazolam, remimazolam besylate did not increase the risk of postoperative cognitive dysfunction in elderly patients, consistent with previous trials (Tan et al., 2022). In this study, both MMSE and MoCA were used to assess postoperative cognitive dysfunction. The MMSE is widely recognized as a brief cognitive assessment tool for measuring cognitive impairment (Arevalo-Rodriguez et al., 2015). However, multiple systematic reviews and meta-analyses have shown that MoCA is more sensitive than MMSE in detecting subtle changes in cognitive function (Arevalo-Rodriguez et al., 2015; Abd Razak et al., 2019; Ciesielska et al., 2016). Compared to the MMSE, the MoCA involves more complex tasks, such as a larger word count, fewer learning trials, and a longer delay before the memory recall test (Nasreddine et al., 2005). These make MoCA scores more influenced by education and cognitive ability. We selected the MMSE for preliminary screening and the MoCA for a more detailed assessment of cognitive function.

Notably, although the surgery and sedation duration in our trial were longer, the total administered dose was lower. Additionally, remimazolam besylate did not increase the incidence of postoperative delirium or adversely impact patient anxiety. Furthermore, remimazolam besylate had positive effects on sleep rhythm, subjective wellbeing, comfort, and patient cooperation during follow-up visits. Satisfaction survey of the attending anesthesiologists yielded results consistent with previous studies (Chen et al., 2020). Moreover, the attending physicians expressed a willingness to explore the remimazolam dosing regimen further in different procedures.

In terms of safety, no hemodynamic deterioration was observed in the remimazolam group, although HR and MAP showed significant differences compared to the control group. However, all vital signs were within the safe range, which positively impacted the anesthesia experience of the patients. The use of vasoactive drugs and the incidence of postoperative adverse effects, including nausea, vomiting, dizziness, drowsiness, headache, and gloss coma, were similar to those in the saline group. Notably, the incidence of chills was significantly lower in the remimazolam group compared to the saline group. Increased muscle activity during anesthesia recovery can raise oxygen consumption by up to five times. Hypoxemia, lactic acidosis, and hypercarbia can complicate anesthesia recovery, particularly in patients already at risk for hypoxemia due to other factors. Even without these complications, patients often experience discomfort due to shivering, and prevention of this syndrome is highly valued (Crossley, 1995). In a phase III trial comparing the efficacy and safety of remimazolam and propofol for colonoscopy, the incidence of treatment-emergent adverse events was lower in the remimazolam group (49.48% vs. 68.42%). However, dizziness (23.71%) and gait disturbance (26.29%) were the primary adverse effects observed. A key reason for this discrepancy may be that, unlike in gastroscopy, where patients experience varying degrees of mechanical trauma, sedation was initiated after satisfactory analgesia was determined from the neuraxial anesthesia in the present study. Consequently, when remimazolam was used for sedation during neuraxial anesthesia procedures, fewer adverse effects occurred because the dose of the sedative drug was significantly lower. Moreover, we selected patients in good physical health and employed a standardized body weight dosing regimen of 0.2 mg·kg^−1^·h^−1^ remimazolam administered via continuous infusion during the maintenance period of sedation.

The correlation between BIS and MOAA/S is shown in Figure 3D. However, the MOAAS score is actually used as the leading indicator for evaluating the sedation levels because evidence suggests that BIS is more suitable for monitoring sedation with propofol (Zhao et al., 2023). In benzodiazepine-based sedation therapy, the BIS index often remains high and can be influenced by other drugs, including anesthetics (Ibrahim et al., 2001). At the same time, the assessment of the BIS index can be influenced by electromyographic (EMG) activity. Literature reports two cases where the BIS index failed to measure anesthesia depth accurately: one case saw a paradoxical increase in the BIS index with rising propofol concentration, correlating with increased myoelectric activity, while in another case, administering a non-depolarizing muscle relaxant decreased the BIS index value despite a constant anesthetic concentration (Bruhn et al., 2000). Consequently, the BIS index may not be fully applicable for evaluating sedation levels in patients undergoing neuraxial anesthesia (Bruhn et al., 2000), warranting further investigation in future clinical trials.

In addition, there was a statistically significant difference in satisfaction between patients and attending anesthesiologists, which we believe serves as supportive evidence for the use of remimazolam in such anesthesia procedures. Through discussions with the attending anesthesiologists involved in clinical drug administration and observation, we learned that anesthesiologists’ satisfaction is primarily influenced by the patient’s level of cooperation during surgery and the occurrence of adverse events. On the other hand, patient satisfaction is more closely related to the degree of tension and anxiety experienced during the surgery and anesthesia, as well as whether they were affected by fear of the procedure. Additionally, postoperative adverse reactions such as nausea, vomiting, and shivering also play a role in patient satisfaction.

Although our dosing protocol was designed specifically for sedation during neuraxial anesthesia in elderly patients, it may also serve as a reference for conscious sedation protocols in elderly patients in other contexts. However, our study had several limitations. First, our study enrolled patients in relatively good health, with well-treated comorbidities and on long-term, regular treatment regimens. Patients with obesity and severe comorbidities were excluded; therefore, in clinical practice, individualized dosing should be considered on a case-by-case basis, because our research findings do not have universal applicability to the above two groups of patients. Second, the concurrent use of opioids and benzodiazepines might exert a synergistic effect that deepens the sedation level. In our protocol, sedation procedures were performed after achieving satisfactory neuraxial anesthesia with promising anesthetic effects, without the use of opioids. Consequently, we could not evaluate the impact of remimazolam besylate in conjunction with opioids and can only offer a reference range for appropriate dosing in elderly patients. Third, different types of surgery pose varying risks for the development of POD. However, the type of surgery was not included as one of the screening criterions in this study, representing a limitation. Fourth, In our study, we adopted a single-blind method where the participants were unaware of their group assignment. Although the interventions administered to the experimental and control groups appeared identical, the anesthesiologists could not be blinded due to the noticeable differences in clinical effects. However, we ensured that the postoperative nursing staff and follow-up evaluators remained unaware of the participants’ group assignments. While this approach mitigated significant bias in the experimental results caused by the lack of blinding to some extent, we acknowledge that the study outcomes may still have been subtly influenced as a result. Finally, regarding the impact of remimazolam on patients’ postoperative cognitive function, we only conducted statistical analyses on short-term outcomes (the morning and afternoon of postoperative day 2, as well as postoperative day 7). Data on its effects on patients’ long-term cognitive function are lacking. Therefore, future studies need to refine and further explore this aspect.

Although spinal anesthesia alone can help patients undergo surgery, administering remimazolam for sedation is more suitable for clinical practice. This preference is based on several factors. First, it results in more stable intraoperative vital signs, such as blood pressure. Second, the use of remimazolam significantly reduces the incidence of perioperative shivering. Lastly, patient satisfaction is higher, aligning with the principles of comfort-focused healthcare. These benefits make remimazolam a valuable option for enhancing both patient outcomes and the overall quality of perioperative care.

## 5 Conclusion

In conclusion, the initial sedation dose of remimazolam besylate in non-general anesthesia surgeries for elderly patients is 5.38 mg, with maintenance dose of 0.223 mg·kg^−1^·h^−1^. Remimazolam besylate does not cause significant hemodynamic fluctuations, nor does it increase the risk of postoperative cognitive dysfunction or delirium. Additionally, it improves sleep quality and enhances the overall anesthesia experience for patients.

## Data Availability

The original contributions presented in the study are included in the article/Supplementary Material, further inquiries can be directed to the corresponding authors.

## Abbreviations

95%CI, 95% confidence interval; ASA, American Society of Anesthesiologists; BMI, body mass index; CONSORT, Consolidated Standards of Reporting Trials; PSQI, Pittsburgh Sleep Quality Index; HAMA, Hamilton Anxiety Scale; Nu-DESC, Nursing Delirium Screening Scale; MMSE, Mini-Mental State Examination; MoCA, Montreal Cognitive Assessment; T-MoCA, Telephone-MoCA; BIS, bispectral index; MOAA/S, Modified Observer Alertness/Sedation Assessment; MAP, Mean arterial pressure; HR, Heart rate; ED_50_, 50% effective dose; ED_95_, 95% effective dose.

## References

[B1] Abd Razak M. A. Ahmad N. A. Chan Y. Y. Kasim N. M. Yusof M. Ghani M. (2019). Validity of screening tools for dementia and mild cognitive impairment among the elderly in primary health care: a systematic review. Public Health 169, 84–92. 10.1016/j.puhe.2019.01.001 30826688

[B2] Aldecoa C. Bettelli G. Bilotta F. Sanders R. D. Audisio R. Borozdina A. (2017). European Society of Anaesthesiology evidence-based and consensus-based guideline on postoperative delirium. Eur. J. Anaesthesiol. 34, 192–214. 10.1097/eja.0000000000000594 28187050

[B3] Arevalo-Rodriguez I. Smailagic N. Figuls M. R. I. Ciapponi A. Sanchez-Perez E. Giannakou A. (2015). Mini-Mental State Examination (MMSE) for the detection of Alzheimer's disease and other dementias in people with mild cognitive impairment (MCI). Cochrane Database Syst. Rev. 2015, CD010783. 10.1002/14651858.CD010783.pub2 25740785 PMC6464748

[B4] Bae M. I. Bae J. Song Y. Kim M. Han D. W. (2024). Comparative analysis of the performance of electroencephalogram parameters for monitoring the depth of sedation during remimazolam target-controlled infusion. Anesth. Analg. 138, 1295–1303. 10.1213/ane.0000000000006718 38051672

[B5] Bruhn J. Bouillon T. W. Shafer S. L. (2000). Electromyographic activity falsely elevates the bispectral index. Anesthesiology 92, 1485–1487. 10.1097/00000542-200005000-00042 10781298

[B6] Chen S. H. Wang J. Xu X. H. Huang Y. G. Xue S. F. Wu A. S. (2020). The efficacy and safety of remimazolam tosylate versus propofol in patients undergoing colonoscopy: a multicentered, randomized, positive-controlled, phase III clinical trial. Am. J. Transl. Res. 12, 4594–4603. 32913533 PMC7476156

[B7] Chen S. H. Yuan T. M. Zhang J. Bai H. Tian M. Pan C. X. (2021). Remimazolam tosilate in upper gastrointestinal endoscopy: a multicenter, randomized, non-inferiority, phase III trial. J. Gastroenterol Hepatol 36, 474–481. 10.1111/jgh.15188 32677707

[B8] Ciesielska N. Sokolowski R. Mazur E. Podhorecka M. Polak-Szabela A. Kedziora-Kornatowska K. (2016). Is the Montreal Cognitive Assessment (MoCA) test better suited than the Mini-Mental State Examination (MMSE) in mild cognitive impairment (MCI) detection among people aged over 60? Meta-analysis. Psychiatr. Pol. 50, 1039–1052. 10.12740/pp/45368 27992895

[B9] Coursin D. B. Coursin D. B. Maccioli G. A. (2001). Dexmedetomidine. Curr. Opin. Crit. Care 7, 221–226. 10.1097/00075198-200108000-00002 11571417

[B10] Crossley A. W. (1995). Postoperative shivering: the influence of body temperature. BMJ 311, 764–765. 10.1136/bmj.311.7008.764 7580430 PMC2550783

[B11] Desai V. Chan P. H. Prentice H. A. Zohman G. L. Diekmann G. R. Maletis G. B. (2018). Is anesthesia technique associated with a higher risk of mortality or complications within 90 Days of surgery for geriatric patients with hip fractures? Clin. Orthop. Relat. Res. 476, 1178–1188. 10.1007/s11999.0000000000000147 29601378 PMC6263607

[B12] Doi M. Morita K. Takeda J. Sakamoto A. Yamakage M. Suzuki T. (2020). Efficacy and safety of remimazolam versus propofol for general anesthesia: a multicenter, single-blind, randomized, parallel-group, phase IIb/III trial. J. Anesth. 34, 543–553. 10.1007/s00540-020-02788-6 32417976

[B13] Fechner J. El-Boghdadly K. Spahn D. R. Motsch J. Struys M. Duranteau O. (2024). Anaesthetic efficacy and postinduction hypotension with remimazolam compared with propofol: a multicentre randomised controlled trial. Anaesthesia 79, 410–422. 10.1111/anae.16205 38221513

[B14] Fick D. M. Semla T. P. Steinman M. Beizer J. Brandt N. Dombrowski R. (2019). American geriatrics society 2019 updated AGS beers Criteria® for potentially inappropriate medication use in older adults. Am. Geriatr. Soc. 67, 674–694. 10.1111/jgs.15767 30693946

[B15] Gu H. H. Deng X. Y. Lv Y. Z. Chen Q. Yu W. F. (2019). Preoperational chronic pain impairs the attention ability before surgery and recovery of attention and memory abilities after surgery in non-elderly patients. J. Pain Res. 12, 151–158. 10.2147/jpr.S178118 30643447 PMC6311326

[B16] Guan X. H. Jiao Z. Y. Gong X. F. Cao H. Y. Liu S. S. Lan H. M. (2021). Efficacy of pre-treatment with remimazolam on prevention of propofol-induced injection pain in patients undergoing abortion or curettage: a prospective, double-blinded, randomized and placebo-controlled clinical trial. Drug Des. Devel Ther. 15, 4551–4558. 10.2147/dddt.S334100 PMC8576108 34764637

[B17] Hosseini L. Abolhasanpour N. (2001). Potential roles of exosomes in aging and age-related diseases. Int. J. Aging 1, e22. 10.34172/ija.2023.e22

[B18] Ibrahim A. E. Taraday J. K. Kharasch E. D. (2001). Bispectral index monitoring during sedation with sevoflurane, midazolam, and propofol. Anesthesiology 95, 1151–1159. 10.1097/00000542-200111000-00019 11684984

[B19] Ichijima R. Ikehara H. Maeda T. Sugita T. Horii T. Iwao A. (2022). First dose-ranging study of remimazolam in Japanese patients undergoing gastrointestinal endoscopy: phase II investigator-initiated clinical trial. Dig. Endosc. 34, 1403–1412. 10.1111/den.14365 35612970

[B20] Jensen L. Monnat S. M. Green J. J. Hunter L. M. Sliwinski M. J. (2020). Rural population health and aging: toward a multilevel and multidimensional research agenda for the 2020s. Am. J. Public Health 110, 1328–1331. 10.2105/ajph.2020.305782 32673118 PMC7427233

[B21] Kornstein S. G. Guico-Pabia C. J. Fayyad R. S. (2014). The effect of desvenlafaxine 50 mg/day on a subpopulation of anxious/depressed patients: a pooled analysis of seven randomized, placebo-controlled studies. Hum. Psychopharmacol. 29, 492–501. 10.1002/hup.2427 25196042

[B22] Lee S. S. Chern J. Y. Frey M. K. Comfort A. Lee J. Roselli N. (2021). Enhanced recovery Pathways in gynecologic surgery: are they safe and effective in the elderly? Gynecol. Oncol. Rep. 38, 100862. 10.1016/j.gore.2021.100862 34621945 PMC8479239

[B23] Lim B. G. Lee I. O. (2020). Anesthetic management of geriatric patients. Korean J. Anesthesiol. 73, 8–29. 10.4097/kja.19391 31636241 PMC7000283

[B24] Liu J. L. Yuan W. X. Wang X. L. Royse C. F. Gong M. W. Zhao Y. (2014). Peripheral nerve blocks versus general anesthesia for total knee replacement in elderly patients on the postoperative quality of recovery. Clin. Interv. Aging 9, 341–350. 10.2147/cia.S56116 24600214 PMC3933353

[B25] Mason S. E. Noel-Storr A. Ritchie C. W. (2010). The impact of general and regional anesthesia on the incidence of post-operative cognitive dysfunction and post-operative delirium: a systematic review with meta-analysis. J. Alzheimers Dis. 22 (Suppl. 3), 67–79. 10.3233/jad-2010-101086 20858956

[B26] Miller D. Lewis S. R. Pritchard M. W. Schofield-Robinson O. J. Shelton C. L. Alderson P. (2018). Intravenous versus inhalational maintenance of anaesthesia for postoperative cognitive outcomes in elderly people undergoing non-cardiac surgery. Cochrane Database Syst. Rev. 8, Cd012317. 10.1002/14651858.CD012317.pub2 30129968 PMC6513211

[B27] Mounet B. Choquet O. Swisser F. Biboulet P. Bernard N. Bringuier S. (2021). Impact of multiple nerves blocks anaesthesia on intraoperative hypotension and mortality in hip fracture surgery intermediate-risk elderly patients: a propensity score-matched comparison with spinal and general anaesthesia. Anaesth. Crit. Care Pain Med. 40, 100924. 10.1016/j.accpm.2021.100924 34217841

[B28] Nakanishi T. Sento Y. Kamimura Y. Tsuji T. Kako E. Sobue K. (2021). Remimazolam for induction of anesthesia in elderly patients with severe aortic stenosis: a prospective, observational pilot study. BMC Anesthesiol. 21, 306. 10.1186/s12871-021-01530-3 34872518 PMC8647449

[B29] Nakayama J. Ogihara T. Yajima R. Innami Y. Ouchi T. (2021). Anesthetic management of super-elderly patients with remimazolam: a report of two cases. JA Clin. Rep. 7, 71. 10.1186/s40981-021-00474-4 34528145 PMC8442815

[B30] Nasreddine Z. S. Phillips N. A. Bédirian V. Charbonneau S. Whitehead V. Collin I. (2005). The montreal cognitive assessment, MoCA: a brief screening tool for mild cognitive impairment. J. Am. Geriatr. Soc. 53, 695–699. 10.1111/j.1532-5415.2005.53221.x 15817019

[B31] Needham M. J. Webb C. E. Bryden D. C. (2017). Postoperative cognitive dysfunction and dementia: what we need to know and do. Br. J. Anaesth. 119, I115–I125. 10.1093/bja/aex354 29161395

[B32] Olotu C. (2021). Anesthesia for the elderly: a narrative review. Minerva Anestesiol. 87, 1128–1138. 10.23736/S0375-9393.21.15388-X 33938679

[B33] Pambianco D. J. Borkett K. M. Riff D. S. Winkle P. J. Schwartz H. I. Melson T. I. (2016). A phase IIb study comparing the safety and efficacy of remimazolam and midazolam in patients undergoing colonoscopy. Gastrointest. Endosc. 83, 984–992. 10.1016/j.gie.2015.08.062 26363333

[B34] Pastis N. J. Hill N. T. Yarmus L. B. Schippers F. Imre M. Sohngen W. (2022). Correlation of vital signs and depth of sedation by modified observer's assessment of alertness and sedation (MOAA/S) scale in bronchoscopy. J. Bronchology Interv. Pulmonol. 29, 54–61. 10.1097/lbr.0000000000000784 34238838

[B35] Rex D. K. Bhandari R. Desta T. Demicco M. P. Schaeffer C. Etzkorn K. (2018). A phase III study evaluating the efficacy and safety of remimazolam (CNS 7056) compared with placebo and midazolam in patients undergoing colonoscopy. Gastrointest. Endosc. 88, 427–437. 10.1016/j.gie.2018.04.2351 29723512

[B36] Rex D. K. Bhandari R. Lorch D. G. Meyers M. Schippers F. Bernstein D. (2021). Safety and efficacy of remimazolam in high risk colonoscopy: a randomized trial. Dig. Liver Dis. 53, 94–101. 10.1016/j.dld.2020.10.039 33243567

[B37] Rundshagen I. (2014). Postoperative cognitive dysfunction. Dtsch. Arztebl Int. 111, 119–125. 10.3238/arztebl.2014.0119 24622758 PMC3959222

[B38] Sheng X. Y. Liang Y. Yang X. Y. Li L. E. Ye X. Zhao X. (2020). Safety, pharmacokinetic and pharmacodynamic properties of single ascending dose and continuous infusion of remimazolam besylate in healthy Chinese volunteers. Eur. J. Clin. Pharmacol. 76, 383–391. 10.1007/s00228-019-02800-3 31873765

[B39] Shi M. Chen J. Liu T. X. Dai W. X. Zhou Z. Chen L. F. (2022). Protective effects of remimazolam on cerebral ischemia/reperfusion injury in rats by inhibiting of NLRP3 inflammasome-dependent pyroptosis. Drug Des. Devel Ther. 16, 413–423. 10.2147/dddt.S344240 PMC8863189 35210755

[B40] Skvarc D. R. Berk M. Byrne L. K. Dean O. M. Dodd S. Lewis M. (2018). Post-Operative Cognitive Dysfunction: an exploration of the inflammatory hypothesis and novel therapies. Neurosci. Biobehav Rev. 84, 116–133. 10.1016/j.neubiorev.2017.11.011 29180259

[B41] Sneyd J. R. Rigby-Jones A. E. (2020). Remimazolam for anaesthesia or sedation. Curr Opin Anesth. 33, 506–511. 10.1097/aco.0000000000000877 32530890

[B42] Tan Y. J. Ouyang W. Tang Y. Z. Fang N. Fang C. Quan C. X. (2022). Effect of remimazolam tosilate on early cognitive function in elderly patients undergoing upper gastrointestinal endoscopy. J. Gastroenterol. Hepatol. 37, 576–583. 10.1111/jgh.15761 34907594 PMC9303590

[B43] Tran T. T. Beutler S. S. Urman R. D. (2019). Moderate and deep sedation training and pharmacology for nonanesthesiologists: recommendations for effective practice. Curr. Opin. Anesthesiol. 32, 457–463. 10.1097/aco.0000000000000758 31219870

[B44] Urvoy B. Aveline C. Belot N. Catier C. Beloeil H. (2021). Opioid-free anaesthesia for anterior total hip replacement under general anaesthesia: the observational prospective study of opiate-free anesthesia for anterior total hip replacement trial. Br. J. Anaesth. 126, E136–E139. 10.1016/j.bja.2021.01.001 33549323

[B45] Wesolowski A. M. Zaccagnino M. P. Malapero R. J. Kaye A. D. Urman R. D. (2016). Remimazolam: pharmacologic considerations and clinical role in anesthesiology. Pharmacotherapy 36, 1021–1027. 10.1002/phar.1806 27496519

[B46] Xiao Q. X. Liu Q. Deng R. Gao Z. W. Zhang Y. (2020). Postoperative cognitive dysfunction in elderly patients undergoing hip arthroplasty. Psychogeriatrics 20, 501–509. 10.1111/psyg.12516 31976614

[B47] Xie H. Y. Lu F. Liu W. L. Wang E. F. Wang L. F. Zhong M. L. (2021). Remimazolam alleviates neuropathic pain via regulating bradykinin receptor B1 and autophagy. J. Pharm. Pharmacol. 73, 1643–1651. 10.1093/jpp/rgab080 34061162

[B48] Zhang S. Y. Wang J. G. Ran R. Peng Y. C. Xiao Y. (2022). Efficacy and safety of remimazolam tosylate in hysteroscopy: a randomized, single-blind, parallel controlled trial. J. Clin. Pharm. Ther. 47, 55–60. 10.1111/jcpt.13525 34655087

[B49] Zhang X. Q. Li S. Liu J. (2021). Efficacy and safety of remimazolam besylate versus propofol during hysteroscopy: single-centre randomized controlled trial. BMC Anesthesiol. 21, 156. 10.1186/s12871-021-01373-y 34016045 PMC8135983

[B50] Zhao Q. Wan H. Pan H. Xu Y. Q. (2024). Postoperative cognitive dysfunction-current research progress. Front. Behav. Neurosci. 18, 1328790. 10.3389/fnbeh.2024.1328790 38357422 PMC10865506

[B51] Zhao T. Y. M. Chen D. Sun H. Xu Z. X. Lyu S. Wang T. (2022). Moderate sedation with single-dose remimazolam tosilate in elderly male patients undergoing transurethral resection of the prostate with spinal anesthesia: a prospective, single-arm, single-centre clinical trial. BMC Anesthesiol. 22, 247. 10.1186/s12871-022-01788-1 35927618 PMC9351252

[B52] Zhao T. Y. M. Chen D. Xu Z. X. Wang H. L. Sun H. (2023). Comparison of bispectral index and patient state index as measures of sedation depth during surgeries using remimazolam tosilate. BMC Anesthesiol. 23, 208. 10.1186/s12871-023-02172-3 37322424 PMC10268360

