# Supplementary material for: The impact of remimazolam sedation during neuraxial anesthesia on perioperative cognitive function in elderly patients: a multicenter randomized controlled study
Source: Front Pharmacol. 2025 Apr 28;16:1504813. doi: 10.3389/fphar.2025.1504813 (PMC12066749; doi:10.3389/fphar.2025.1504813)
Supplement: Supplementary file 1 [file Table1.docx]

**Supplementary Table S1 Patients and Researcher satisfaction.**

|  | Remimazolam  (n=160) | Saline  (n=78) | P |
| --- | --- | --- | --- |
| **Patient**  satisfaction score |  |  |  |
| Day 2 morning | 9.08 (9.02 to 9.15) | 8.10 (7.81 to 8.40) | **<0.001** |
| Day 2 afternoon | 9.08 (9.00 to 9.15) | 8.06 (7.76 to 8.37) | **<0.001** |
| Day 7 | 9.09 (9.02 to 9.17) | 8.28 (8.12 to 8.45) | **<0.001** |
| **Researcher** satisfaction score |  |  |  |
| Day 2 morning | 9.13 (9.06 to 9.19) | 8.15 (7.90 to 8.41) | **<0.001** |
| Day 2 afternoon | 9.12 (9.04 to 9.19) | 8.13 (7.86 to 8.40) | **<0.001** |
| Day 7 | 9.15 (9.08 to 9.22) | 8.27 (8.10 to 8.43) | **<0.001** |

Data are mean (95% CI). CI, confidence interval.
