# Supplementary material for: The impact of remimazolam sedation during neuraxial anesthesia on perioperative cognitive function in elderly patients: a multicenter randomized controlled study
Source: Front Pharmacol. 2025 Apr 28;16:1504813. doi: 10.3389/fphar.2025.1504813 (PMC12066749; doi:10.3389/fphar.2025.1504813)
Supplement: Supplementary file 2 [file Table2.docx]

**Supplementary Table S2 Side-effect.**

| Side-effect | Remimazolam  (n=160) | Saline  (n=78) | P-value |
| --- | --- | --- | --- |
| Phenylephrine | 5 (3.13%) | 5 (6.41%) | 0.236 |
| Atropine | 6 (3.75%) | 4 (5.13%) | 0.619 |
| Dopamine | 7 (4.38%) | 6 (7.69%) | 0.291 |
| Metaraminol Bitartrate | 6 (3.75%) | 3 (3.85%) | 0.791 |
| Methoxamine | 4 (2.50%) | 3 (3.85%) | 0.564 |
| Ephedrine | 4 (2.50%) | 4 (5.13%) | 0.291 |
| Nausea | 2 (1.25%) | 1 (1.28%) | 0.983 |
| Vomiting | 1 (0.63%) | 1 (1.28%) | 0.602 |
| Chills | 0 (0) | 13 (16.67%) | **<0.001** |
| Drowsiness | 2 (1.25%) | 0 (0) | 0.321 |
| Headache | 1 (0.63%) | 0 (0) | 0.484 |
| Dizzy | 2 (1.25%) | 0 (0) | 0.321 |
| Gloss coma | 2 (1.25%) | 0 (0) | 0.321 |

The data are reported as n (%). The chi-square test was used.
